# Mrs. Winslow’s Soothing Syrup—a Poison

**Published:** 1872-06

**Authors:** W. F. M’Nutt

**Affiliations:** San Francisco


*Mrs. Winslow's Soothing Syrup—A Poison.* By W. F. M'Nutt, M.D., San Francisco.

My attention was first called to the baneful effects and the enormous consumption of this nostrum, by an article in the November (1869) number of the *California Medical Gazette*, by Dr. Murray,

U.S.A. Dr. Murray had been called to see a child aged six months, apparently in a dying condition from the effects of some narcotic poison. He found that this soothing syrup was the only medicine which had been administered, and of it the child had taken two teaspoonfuls within ten hours. There was remaining in the vial from which the two teaspoonfuls had been taken, ten drachms, which yielded, on analysis by a skillful chemist, nearly one grain of morphia and other opium alkaloids to the ounce of syrup. "The specimen of Soothing Syrup analyzed was made by Curtis & Perkins, of New York, who are the only manufacturers."

On the 7th of February, Mrs. W. came into my office with a child five months old in her arms, which, she said, was very sick; that it slept constantly, and would not nurse or move for several days. The child was breathing heavily, and its pupils were closely contracted. I asked if the child had been taking opium; she replied that it had taken nothing but soothing syrup. She said that on the 5th, two days before, the child was restless and its bowels costive, and that a neighbor had advised her to give it a teaspoonful of soothing syrup, saying it was excellent to regulate the bowels. (She had previously given the syrup in small doses.) She administered the syrup twice during the day, a teaspoonful each time; the child slept heavily all night, and would not nurse when roused. Not suspecting that the syrup had anything to do with its sleeping, she gave on the 6th, at different times, three teaspoonfuls more. The child refused to nurse when roused. On the 7th she gave it another teaspoonful, before bringing it to my office. I told her that the child was poisoned by morphia, of which soothing syrup contained a large quantity. The mother was surprised and alarmed, having had no idea that there was morphia in soothing syrup.

I ordered brandy and coffee, the bowels to be kept open by injections, and the child to be kept awake as much as possible. The child recovered, but was not able to nurse until the 10th. This is but one of the many instances of poison by this nostrum.

Dr. R. S. Maxwell, my partner, was called to see a child five weeks old, to whom a half a teaspoonful of soothing syrup had been given a few hours previous. The child was already past all help, and died in a few hours. No other medicine had been given.

In my own case, the child five months old had taken two teaspoonfuls on the 5th, three on the 6th, and one on the 7th, making six teaspoonfuls from ten o'clock on the 5th until eight A. M. on the 7th; consequently it got over half a grain of morphia in the space of forty-six hours. As susceptible as children are to the influence of opium, it seems almost impossible that the child could have lived. In fact, we know that it could not have lived, had not the tolerance of the poison been induced by previous doses in lesser quantities. We may add that there are very few children at the age of six months, who would not be poisoned to death, were they to take the syrup as directed, (namely: six months old and upwards, one teaspoonful three or four times a day until free from pain,) unless a tolerance of the drug be induced by its previous administration in small doses. The morphia in a teaspoonful of soothing syrup is equal to about twenty drops of laudanum. Here we have thousands of mothers and nurses, ignorant alike of the ingredients and the effects of this deadly nostrum, directed to give a child, six months old, morphia equal to twenty drops of laudanum, while a physician would not dare to give a child of that age more than three drops.

Dr. Murray, in the article already referred to, says: "I have ascertained that there are about one hundred thousand two-ounce bottles of it sold annually in this city, containing about one hundred and eighty thousand grains of morphia, which are given annually to the babies of this State."

If the babies of this State consume two hundred thousand ounces of soothing syrup, it is but fair to assume that there is seventy-five times that amount used in the whole United States, which would make 15,000,000 ounces of syrup, or about 14,000,000 grains of morphia. Setting aside the direct cost of the nostrum, it would be scarcely possible to estimate the damages which the people of the United States sustain indirectly from its use.

How much the early resort of our youth to tobacco and alcoholic stimulants is due to the previous use of the opium contained in this nostrum is probably not realized. But, that it has much to do with it, any one can believe, who has seen with what avidity the opium eater, when deprived of his opium, will fly to alcohol, ether, hashish, tobacco, or anything that will lull the eternal craving of the appetite for something, other than wholesome food. It would be also impossible to estimate the number of children it sends to the grave before they reach their second year. But that the administration of 14,000,000 grains of morphia annually, to the babies of the United States, by persons ignorant of its effects, must send its thousands, any reasonable person will be inclined to grant. But a still graver question presents itself, namely: How much of the physical disease, of the drunkenness, of the degradation and of the vice, and how many of the weakened intellects, are due to the use of the soothing syrup in infancy? Probably enough to make it a wiser Legislature that will prohibit the manufacture of any nostrum for children which contains opium, than the Legislature that passes a prohibitory liquor law for the benefit of its adults.—*Pacific Med. and Surg. Journal.*

